# EOLinPLACE: an international research project to reform the way dying places are classified and understood

**DOI:** 10.1177/26323524231222498

**Published:** 2024-02-12

**Authors:** Elizabeth Namukwaya, Andrea Bruno de Sousa, Sílvia Lopes, Dorothea Petra Touwen, Jenny Theodora van der Steen, Emmanuelle Bélanger, Joanna Brooks, Stecy Yghemonos, Kawaldip Sehmi, Barbara Gomes

**Affiliations:** Faculty of Medicine, University of Coimbra, Coimbra, Portugal Department of Medicine, College of Health Sciences, Makerere University, Kampala, Uganda; Faculty of Medicine, University of Coimbra, Coimbra, Portugal; NOVA National School of Public Health, Public Health Research Centre, Comprehensive Health Research Center, CHRC, NOVA University Lisbon, Lisbon, Portugal; Department of Medical Ethics and Health Law, Leiden University Medical Center, Leiden, the Netherlands; Department of Public Health and Primary Care, Leiden University Medical Center, Leiden, the Netherlands; Department of Primary and Community Care and Radboudumc Alzheimer Center, Radboud University Medical Center, Nijmegen, the Netherlands; Center for Gerontology and Healthcare Research, Brown University School of Public Health, Providence, RI, USA; Population Health and Palliative Medicine, Master of Health Services Administration, University of Kansas School of Medicine, Kansas City, KS, USA; Eurocarers, Brussels, Belgium; International Alliance of Patients’ Organizations, London, UK; Faculty of Medicine, University of Coimbra, Pólo III, Sub-Unidade 3, Azinhaga de Santa Comba, Coimbra 3000-548, Portugal Cicely Saunders Institute of Palliative Care, Policy and Rehabilitation, King’s College London, London, UK

**Keywords:** classification, death, death certificates, mortality, palliative care, patient preference, terminal care

## Abstract

**Background:**

Whenever possible, a person should die where they feel it is the right place to be. There is substantial global variation in home death percentages but it is unclear whether these differences reflect preferences, and there are major limitations in how the place of death is classified and compared across countries.

**Objectives:**

EOLinPLACE is an international interdisciplinary research project funded by the European Research Council aiming to create a solid base for a ground-breaking international classification tool that will enable the mapping of preferred and actual places towards death.

**Design:**

Mixed-methods observational research.

**Methods and analysis:**

We combine classic methods of developing health classifications with a bottom-up participatory research approach, working with international organizations representing patients and informal carers [International Alliance of Patients’ Organizations (IAPO) and Eurocarers]. First, we will conduct an international comparative analysis of existing classification systems and routinely collected death certificate data on place of death. Secondly, we will conduct a mixed-methods study (ethnography followed by longitudinal quantitative study) in four countries (the Netherlands, Portugal, Uganda and the United States), to compare the preferences and experiences of patients with life-threatening conditions and their families. Thirdly, based on the generated evidence, we will build a contemporary classification of dying places; assess its content validity through focus groups with patients, carers and other stakeholders; and evaluate it in a psychometric study to examine construct validity, reliability, responsiveness, data quality and interpretability.

**Ethics:**

Approved by the ethics committee of the University of Coimbra, Faculty of Medicine (CE-068-2022) and committees in each of the participating countries.

**Discussion:**

The findings will provide a deeper understanding of the diversity in individual end-of-life pathways. They will enable key developments such as measurement of progress towards achievement of preferences when care can be planned. The project will open new directions in how to care for the dying.

**Trial registration:**

Research Registry UIN 9213.

## Background

The places where people die have evolved over the centuries and whilst for most of history the majority of people died at home surrounded by family, this norm began to change in the mid-20th century,^1^ in parallel with the second phase of the epidemiological transition. Hospitals became the locus of medicine and providers of cure for previously serious diseases. The shift from dying at home with family to dying in the hospital alone (called by Ariès the ‘displacement of the site of death’)^2^ occurred steadily over decades, amplified by urbanization and immigration. By the late 1970s and 1980s, in several nations, most of all deaths occurred in hospitals.^3^ In the last three decades, however, the global scenario has changed. The nearly universal hospitalization trend has been replaced by multiple realities, with several countries shifting towards dying in the community. Some began to see a drop in hospital deaths and a rise in home deaths – the United States in the 1980s,^4^ Canada in the 1990s, and China and the United Kingdom in the 2000s.^5^ Others have seen a shift away from hospitals into care/nursing homes, for example, Switzerland, Germany, the Netherlands^6^ and Belgium, especially in the late 1990s and early 2000s; however, in the Netherlands, this shift was reversed in 2015 after extensive reforms to control long-term care expenditures (with budget cuts and closure of long-term care facilities).^7^ A UK projection study published in 2018 warned that if trends continued, the number of deaths in care homes and homes would increase by 108% and 89%, with care homes becoming the most common place of death by 2040.^8^ In other countries, the hospitalization trend persists, for example, Greece, Portugal,^9^ Japan and Korea. Low-income and middle-income regions take global variation in place of death even further. A study by Adair published in 2021 estimated that 53% (95% CI: 51–56) of deaths across 49 countries occurred at home, substantially higher in low-income countries (80%, 95% CI: 77–82) and much lower in high-income countries (27%, 95% CI: 25–30).^10^

It is unclear whether these differences reflect preferences. Our previous research that surveyed the general population in nine countries (seven European and two African),^11–13^ regarding their preferences for place of death revealed that most adults would prefer dying at home in a scenario of advanced illness. Qualitative studies show that being at home makes patients feel empowered and safe.^14,15^ Overall, hospitals are the least preferred places,^14^ although in some cultures and for some populations (e.g. child patients), more people may prefer hospitals, with the belief that the best care is provided there.^16–18^ Also, there have been concerns that when faced with the reality of illness progression towards death and complications, preferences may change and patients may end up dying in hospital. Notwithstanding, there is little evidence on how people’s preferences evolve over time in relation to their experiences of places and this is a critical gap. Preferences may also be shaped by the availability of services in different settings and the perceived quality of care. The COVID-19 pandemic brought other aspects into the equation; avoiding the risk of infection in institutional settings may have led more people to remain at home towards the end of life.^19^ Some countries reported rising home deaths during the pandemic,^20^ although it is unclear yet if this was a global trend and whether or not it will persist.

Cross-national analyses and country comparisons of place of death based on death certificate data constitute important research as they can reveal trends and determinants of where people die at a population level but there are major limitations. Current classifications of places of death are inconsistent and incomplete; this results in a lack of knowledge of how many people die in places that are qualitatively distinct from each other. For example, dying in a hospital palliative care unit can be very different from dying in another hospital service (e.g. emergency department) or in a hospital corridor – all usually classified as ‘hospital’. Also, dying in a care home or the home of a relative or friend is not necessarily the same as dying at one’s own home. The home of a relative or friend is also one of the least preferred places to die, particularly in Africa.^12^ Several countries record all these places under ‘home’.

Lastly, much of what is known about dying places is focused on the endpoint – the place of death^3,21^ with not much attention to the place of care, and yet the latter often determines the former. Patients can move between settings, particularly within the last few months of life. To date, little is known about the pathways and last transitions leading someone to die in a specific place. However, interest in this subject has increased following research from the United States with a random sample of 848,303 Medicare beneficiaries aged 66 years and over, showing that whilst deaths in acute care hospitals decreased, intensive care unit (ICU) use and transitions between places in the last 90 days of life increased (from 2000 to 2009).^22^ This study warned of finer differences in care patterns that we are unable to discern if we only examine the place of death. However, an important research question remains unanswered: are these transitions aligned with the preferences of patients and their family members? To the best of our knowledge, no study yet mapped transitions in relation to patient and family preferences over time towards death.

To meaningfully compare preferred and actual places towards death, we need a reliable and accurate common metric. A major global reform and a contemporary classification are opportunities to harmonize information recording so that comparable data can be obtained across countries. Now is the time to pursue this reform, in light of the fast-growing palliative care need in a post-pandemic world.

## Objectives

We are driven by the vision of reforming the way dying places are classified and understood, refining and shifting the focus from the endpoint (place of death) to the pathway that precedes it. We aim to develop a strong foundation for a pioneering international classification tool that maps preferred and actual places towards death grounded on what they mean for individuals (beyond a purely physical or medical view). To achieve this aim, our objectives are as follows:

1. To develop an international classification of dying places (ICP) that fully captures the diversity of places that are meaningful for individuals. To achieve this, we will cross-link data on the preferences and experiences of patients with life-threatening chronic conditions and their families with current classifications and data;
2. To examine whether an ICP can be robustly applied cross-nationally to map differences in preferences, places of care and places of death;
3. To deepen understanding of the different pathways and transitions that are meaningful for individuals in caring environments, and their influencing factors.

**Figure 1.**
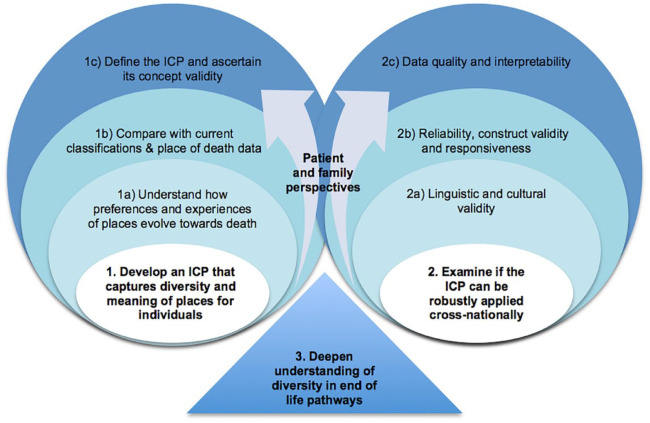
EOLinPLACE implementation plan.

Figure 1 shows a schematic view of the project implementation plan. This consists of the two objectives (1 and 2, white circles) each composed of specific tasks (blue circles), and the final third objective (3, blue triangle) that results from the integration of all project findings. A participatory research approach will be employed to ensure patient and family perspectives are at the heart of the ICP and their involvement will gradually increase along the implementation plan, through contributions in the Project Advisory Group to collaborative working in tasks and focus groups (arrows).

## Methods

### Study design

We will combine classic methods of developing health classifications, guided by recommendations from the United Nations on classifications and from the World Health Organization on how to develop health classifications.^23,24^ We will break conventional research processes and adopt a bottom-up participatory research approach (novel in the development of health classifications), by handing power to patient and family representatives, carrying out the research *with* them rather than *on* them.^25^ This approach is increasingly used in social and health sciences to actively involve target populations in defining the problem, generating analyses and having power over choices and on their implementation.

Different studies will be conducted using various methods in a phased manner starting with Study 1 to Study 3. First, we will conduct an international comparative analysis of existing classifications and routinely collected death certificate/registration data on place of death (Study 1). Second, we will conduct a novel mixed-methods study (Study 2) in four countries covering existing target variation (the Netherlands, Portugal, Uganda and the United States), to compare the preferences and experiences of patients with life-threatening conditions and their families. This will consist of ethnography (Study 2.1) followed by a longitudinal quantitative study (Study 2.2). Based on the generated evidence, we will build a contemporary classification of dying places and undertake a validation study (Study 3). This will start with an assessment of content validity in focus groups with patients, carers and other stakeholders (Study 3.1). We will then test the classification in varied settings in a psychometric study to examine construct validity, reliability, responsiveness, data quality and interpretability (Study 3.2). All data will be integrated to deepen understanding of different end-of-life pathways in caring environments and what influences them.

### Country case studies

**Figure 2.**
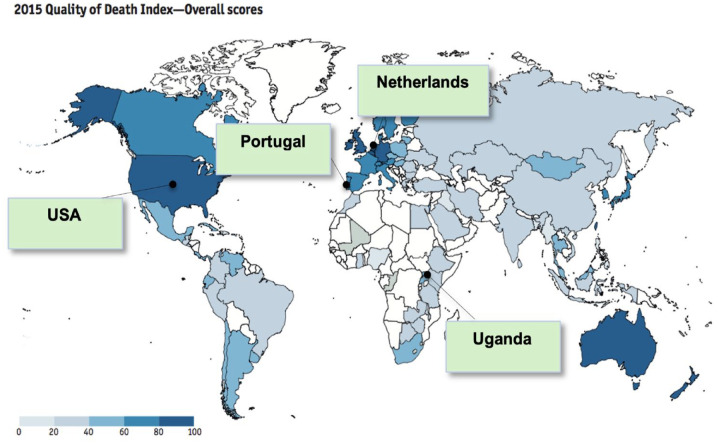
Country case studies. Source: Economist Intelligence Unit (2015).^26^

The four countries – the Netherlands, Portugal, Uganda and the United States – were purposively selected to capture variation in five specific criteria: (i) different ranks in the Quality of Death Index (Figure 2); (ii) focused on European variation but include one most and one least developed non-European country with proven research capacity on end of life care studies; (iiii) low and high preference for dying at home (e.g. 51% in Portugal *versus* 84% in the Netherlands in an earlier cross-country population survey)^11,14^; (iv) different place of death trends^4–6,9^; (v) rudimentary and finer place of death classifications (Table 1).

**Table 1.** Place of death classifications used in death certificates in four countries.

Netherlands	Portugal	Uganda	United States
Hospital Psychiatric institution Nursing home Care home Hospice Home Other (specify) Unknown	Health facility - Hospitals • Inpatient ward • Emergency department • Intensive care unit • Other - Local Health Units/health centres - Other Home^a^ Other (specify) Unknown	Recorded in free text^b^	Hospital - Emergency room/outpatient - Dead on arrival Other than hospital - Hospice facility - Nursing home/long-term care facility - Decedent’s home - Other (specify)

a Includes home and care homes/nursing homes.

b No instructions are given about what to write but doctors often use home, hospital, clinic, on the way to hospital, on the road – accident (according to the information given by local doctors).

Each study is now described in detail.

### Study 1: International comparative analysis of the place of death

We will conduct an international comparative analysis of the place of death of existing classification systems and routinely collected death certificate data on place of death. This will be done in two ways: using aggregated data and individual anonymized data. Preliminary work for this study includes systematically reviewing the literature about the dying places preferred by patients and their family members^27^ and about the cross-national commonalities and differences in place of death from international comparative death certificate studies.

#### Aggregate data analysis

Data on place of death derived from death certificates of all individuals of all ages who died from all causes from 1 January 2012 to 31 December 2021 in national territory will be sought from national statistics offices/health agencies/vital registries of a selection of 47 countries. These included all 27 European Union countries (where the work originated) and 20 additional countries chosen to cover variation in United Nations Regions and the Quality of Death and Dying Index 2021.^28,29^ We will analyse the distribution between different places, how it varied over time and the level of missing data. In addition, we will survey key research groups working on place of death in a subsample of countries to gather their views as data users about the systems used to classify place of death in the same 10 years.

#### Individual data analysis

In the Netherlands, Portugal, the United States and Uganda, we will access and analyse individual anonymized data on the place of death and associated factors (including gender, age and cause of death, among others) derived from death certificates of all individuals who died from all causes from 1 January 2012 to 31 December 2021. We will examine trends, with an emphasis on sudden changes in the distribution of place of death, including comparisons pre- and post-COVID-19. We will describe changes in the recording/coding of the place of death and missing data, studying their association with individual characteristics of the deceased (e.g. sociodemographic, cause and period of death). Whenever possible, we will use multivariate analysis to identify sub-groups (e.g. specific age and disease groups) with increased risk of missing/unspecific coding of place of death. If there were changes in recording/coding during the study period, we will examine their impact and compare pre- and post-change data for the whole population and relevant sub-groups.

### Study 2: Mixed-methods study of end-of-life pathways

To deepen our understanding of the different pathways and transitions in places that are meaningful for individuals and their influencing factors within and between the four countries, we will undertake a sequential exploratory mixed-methods study, first using ethnography followed by a quantitative longitudinal study.

#### Ethnography

We will draw on three methods used in systematic comparative ethnography: review of documents, interviews and non-participant observation (Table 2). Systematic comparative ethnography makes use of ethnography to collect data that are both rich and systematically comparable by following a single study design developed with consideration of the specific research questions and contexts of the research. The same methods are applied across sites and a systematic analysis follows. The design has been successfully applied in fields of public health (e.g. to study AIDS, marriage and work; to evaluate the impact of measles and polio eradication initiatives).^30^

**Table 2.** Methods used in systematic comparative ethnography.

*Review of documents*: The local research team will collect and code as many documents as possible that determine the regulation or assessment of place of care and death from a standardized list (e.g. death certificate templates and instruction manuals, policy documents on end-of-life care at national and regional levels). This will enable us to capture how the notion of place of care and death at the end of life is integrated into policy and practice documents.
*Interviews*: Interviews will be carried out with national- and regional-level health policy officers, ground-level health and social care professionals and representatives from patient and family carer organizations, among others (around 25 individuals in each country, 100 in total). For each, researchers will follow an interview topic guide that will be the same across sites to achieve insight into the conceptions of place of care and death of different stakeholders in the field, their impact on practice and needs for revision, in both policy and practice. However, within an ethnographic approach, interviews are open and in-depth. Therefore, some topics may not be relevant during certain interviews, while others are of particular importance and lead to unexpected findings. Flexibility will allow capturing context-specific information from the different countries.
*Non-participant observation*: This will involve closely following over time a small number of families facing a life-threatening chronic illness (around 5 in each country, 20 in total) from whom in-depth case studies will be developed to gain insight into actual and preferred places of care and death. We will recruit from hospital services, working in close collaboration with the care staff that will identify eligible patients. The eligibility criteria will be the same as for the longitudinal and validation studies [but conducted purposively, aiming to cover, within the small number of families, as much diversity as possible in the pathways and the places where the patient may be (living at home, care home, hospitalized, etc.), disease groups and ages (minors, adults)]. This will require some flexibility, as recruitment decisions will be made progressively, depending on attrition, the number of families the researchers can take on to attain the required data that are of sufficient quality, and the emerging findings, as analysis will progress concurrently to data collection. The aim is to document different trajectories regarding place of care and death so it is expected the researchers will conduct observation of end-of-life care in the different places people may receive care from.

At the same time, ethnography is an iterative, cumulative process and the benefit of the openness this offers should not be underestimated as it accommodates the exploratory nature and the complexity of a topic as the diversity and dynamics of place of care and death. This approach requires flexibility in the methods used, the number of participants recruited and the number and frequency of interviews.

Observation of daily life situations and interactions between patients, relevant others and care professionals will be done. Informal conversations will be held with patients and their family carers on an *ad hoc* basis depending on the natural flow of events. As relevant information emerges outside the boundaries of the interview situation, they are a valuable source of insight and follow-up. Informal conversations will form the main way to connect with those people that make up the care network around the patient and family carer such as care staff and broader social network (e.g. family or friends). Notebooks will be used to record fieldnotes from observations and informal conversations during, or as soon as possible after the events and will also be used to record context-specific information and cultural aspects from the different countries. Other channels such as phone calls and messaging may be used to communicate with patients and family carers as and when appropriate. Online monthly debriefings of the research team will be conducted to facilitate the analysis and ensure that no emerging themes are overlooked. All the fieldwork is expected to be carried out within 1 year.

All researchers involved will be trained in ethnographic methods such as conducting non-participant observation and creating rich fieldnotes, applied to the project. The research will be carried out by native language speakers in each country, supervised by senior research team members assigned to coordinate the study in that country. They will code the documents, fieldnotes and interview transcripts in the original language, applying a common set of codes across all sites (translated into English) for those developed deductively (based on the research questions), allowing more codes to develop inductively (based on the data). The team will come together during and after fieldwork to address the strengths and weaknesses of the data, reflect on what the various stakeholders say in interviews and what we will learn in non-participant observation. This will result in a body of data that is equivalent across countries so we can compare patterns of variation in preferences and places in the four country case studies. The findings will shape how preferences, places of care and place of death are measured in the subsequent longitudinal study and the ICP.

#### Longitudinal quantitative study

We will undertake a longitudinal quantitative study with a planned total sample of 392 families of patients with life-threatening conditions (98 in each of the four countries). This study aims to unravel changes in preferences for dying places over time, map these with actual pathways and identify influencing factors. Families will be followed for 1 year, with the view to capture individual end-of-life pathways towards death.

We postulate that there will be considerable heterogeneity in end-of-life pathways between conditions leading to death and age groups.^31^ We will therefore include child, adolescent and adult patients with one of the following four diseases in advanced stage: (i) heart and cerebrovascular diseases (long-term limitations with intermitting serious episodes), (ii) cancer (short period of evident decline), (iii) dementia (prolonged dwindling in mostly older people, only adults) and (iv) neuromuscular disorders (prolonged dwindling in younger populations). The inclusion of children/adolescents (in all disease groups except dementia) is important because they constitute a major risk group for dying in a hospital for whom we know very little about preferences and end-of-life pathways.^18,32^

Patients will be excluded if their life expectancy is more than 6 months (clinicians’ judgement) or less than 2 weeks (too short for serial interviewing); if they are too ill, stressed or overwhelmed to take part (clinicians’ judgement) and if they are unable to understand or communicate in the local language. Primary family carers will be identified by the patients (or by care staff on their behalf in case of adults with incapacity or minors) as the family member providing the most help with their care. The sampling will not result in any discriminatory practices (e.g. those with auditory problems or those unable to physically sign consent) and it is important to point out that we will not exclude illiterate people but put in place procedures to enable their participation.

We will identify patients from hospital services in four countries: the Coimbra University Hospital Centre in Portugal, the Mulago Hospital Complex in Uganda, the Leiden University Medical Center in the Netherlands and the University of Kansas Medical Center in the United States. Additional sites may be included depending on recruitment needs, time and resources. Medical and/or other care staff at each recruitment site will identify eligible individuals according to our inclusion and exclusion criteria, first approach the patients and their families to present the study and invite them to take part, provide them the consent form with information about the study and ask permission to pass on their contact details to the research team. If they agree, a researcher will contact them to further explain the study, ask if they are willing to take part, clarify any questions they may have, obtain written informed consent and conduct the first interview at the most convenient place for the participants. In the case of patients who are under 18 years of age and people with cognitive impairment resulting in incapacity to provide consent (clinicians’ judgement), parents/legal representatives will be approached first. If the parents/legal representatives agree, the patient will then receive information about the study, adequate to their maturity/capacity of comprehension and their level of awareness of the diagnosis and prognosis (based on the parents/legal representative’s information). Informed consent and assent when applicable, and the first interview follows.

We will respect local law and cultural sensitivities, particularly regarding the ages children should be approached. The assessment of preferences poses specific ethical issues with children/minors. Our approach will be to not assess preferences with children under the age of 6 years in all countries except Uganda where we will not assess preferences with children under the age of 8 years to comply with national law on age for assent (assessment only with parents/guardians) and assess preferences only for place of care with children aged 6–15 (8–15 in Uganda); for those aged 16 and over we will take the same approach as with adults, that is, assess preferences for place of care and for place of death when appropriate and only if they are aware of the prognosis. The preferences for place of care and death of adults who lack capacity will be assessed *via* proxies (their family carers). The findings from the previous studies (i.e. international comparative place of death analysis and ethnography) will inform the categories of place that will be used in the assessment of preferences, places of care and place of death.

Refusal or exclusion of the patient does not necessarily mean the family carer will not be able to take part. If the patient declines or is unable to participate, the family carer can still take part in the study. Likewise, if the family carer declines, the patient can still take part. Notwithstanding, we will seek patient assent for family carer participation and respect the wishes of the patient if they disagree. Participants will be free to withdraw from the study at any time.

There will be a comprehensive face-to-face interview with the patient and the primary family carer separately but on the same day, at baseline, and subsequently at monthly intervals. Short telephone interviews will be completed every 2 weeks at a minimum in between to assess relevant changes. We will examine places of care and the percentage of time that patients spent in various places, numbers and types of transitions and place of death. We will graphically present these data to illustrate patterns of transitions and diversity in end-of-life pathways. We will also examine the most and least preferred places, any changes and the time in which these occur, and alignment with actual places (including death occurrence in the place of choice). Other differences to explore include preferences in ideal *versus* actual circumstances; preferences for place of care *versus* place of death; and patients *versus* family carers (differences and agreement, using paired data from each dyad). We will also examine the relative influence of different factors (such as diagnosis, age, marital status, relationship with the family carer, symptom severity, survival time)^33^ on changes in preferences and actual places of care and death. We will analyse the data both forward and backward from death (the latter will include patients who die during the 1-year study follow-up). Analyses will be adjusted for key characteristics that determine the place of death, and a two-sided *p* value of less than 0.05 will be considered to indicate statistical significance.

The new evidence generated from studies 1 and 2 above will contribute to understanding the dynamics and full spectrum of preferred and actual dying places. It will be triangulated to inform the development of the ICP. We plan to create an easy-to-use classification with a hierarchical structure of exhaustive, mutually exclusive, well-balanced and stable higher and lower-level categories with unique unambiguous descriptions and a consistent relationship between them.

### Study 3: Validation study

#### Focus groups for content validity

After the development of the ICP (based on evidence from studies 1 and 2), we will undertake focus groups to establish its content validity, relevance and comprehensiveness in an environment that stimulates interaction. We will carry out three focus group discussions of 8–10 participants in each of the four countries included. We plan to conduct one group with patient and family carer representatives, one group with care staff representatives (target classification user group) and one group with researchers (including measurement experts) together with policymakers and other key stakeholders. This will result in a total of around 30 participants per country; 120 across the four countries involved. We plan to conduct these discussions physically but should circumstances (e.g. COVID-19, Ebola) prevent face-to-face meetings, the discussions will be held online, *via* Skype/Zoom or any similar platform. On the other hand, in countries or regions where it may be difficult to meet in person due to long distances (e.g. in the United States) focus group discussions will be conducted online.

Since this is a content validity study for a classification tool aimed to be used internationally, we will ensure that the classification is acceptable by stakeholders and is fit for purpose internationally. Therefore, we will also conduct three international online focus groups of participants from other countries totalling 30 participants.

Together with the patient and carer organizations partners in the project [International Alliance of Patients’ Organizations (IAPO) and Eurocarers], we will design a sampling strategy to recruit representatives of local patient and carer organizations, reaching out for rare and uncommon experiences and diseases. Care staff, researchers and other stakeholders will be recruited through key conferences and contacts of our Project Advisory Group members.

Two trained facilitators will follow a focus group guide common to all countries but allowing flexibility to be open-ended and adaptable to context-specific circumstances. We will share the planned categories of the ICP with the participants, and the guide will also aim to discuss the relative importance of each category and the relevance and comprehensiveness of the overall classification. Fieldnotes will be written by the group facilitators and the discussions will be recorded and transcribed (in the original language), coded in English using the same codes across all sites, developed both deductively and inductively, with the analysis taking place concurrently with the focus groups, to inform subsequent groups. Using thematic analysis, we will (i) analyse how the different stakeholders characterize and differentiate dying places, (ii) compare the elicited themes with the planned ICP categories, (iii) identify places that were not anticipated in the previous methods but that emerge from stakeholders’ views and (iv) examine the words they use to describe different places. Finally, the views of patients, families, care staff, researchers, policymakers and others will be triangulated. Based on the themes generated from the qualitative analysis, we will clarify concepts, refine ICP constructs, and if need be add new categories.^34^ This will help us improve the ICP and ensure it is acceptable to all stakeholders, fit for purpose and appropriate for different languages and cultures, achieving objective 1 of the project. Focus groups have provided unique insights into the development of instruments and their content validity from the perspective of people with lived experience, ensuring adequate coverage of all components of the domain of interest.^34^

#### Psychometric study

Following systematic, culture-sensitive and harmonized translations of the ICP from the English version into Dutch and Portuguese following European Organisation for Research and Treatment of Cancer (EORTC) translation procedures,^35^ we will undertake cognitive interviewing and piloting work in one clinical site per country. We will train classification users (care staff members) to assess and record preferences and actual places of care over time (including transitions) as part of their routine clinical practice using the classification for newly referred patients with advanced disease. They will be asked to repeat their assessment regularly, recording time, effort and other demands placed on patients/carers and themselves (respondent and administrative burden), including situations when preference assessment was unsuitable (with reasons why). They will also be asked to record additional socio-demographic and clinical information. The staff will apply the classification to record the place of death if the patient dies during the study period. We will run a pilot phase with five families per country (ideally from referral to death). Additionally, we will conduct cognitive interviews with the care staff members to explore information processing and errors, cultural relevance of the translation, ease of use of the classification and integration in routine care. We will also examine respondent and administrative burden, time to complete and missing data (and their explanations). The analysis will lead to final revisions and an ICP version for further validation.

Following piloting, the main phase will take place in test sites in the four countries (covering adult and paediatric environments). Eligible sites will include hospitals, primary care practices, care homes, hospices or palliative care services (open to further, subject to resources/time availability). Following the piloted procedures, we will train the care staff to integrate the use of the ICP in routine care, assessing newly referred patients with advanced disease and regularly thereafter (same groups as in the longitudinal study). The analysis will be conducted using the data recorded by the professionals, which will be pseudonymized and provided to the research team for analysis. Variables will include preferred and actual places of care and death and additional socio-demographic and clinical information for comparison of groups.

Using the data produced, we will examine the five measurement properties shown in Table 3: (i) reliability, (ii) construct validity, (iii) responsiveness, (iv) data quality and (v) data interpretability; these are standard quality evaluation criteria for health status and quality of life measures that apply to health classifications.^36,37^ Three other measurement properties – content validity, burden (respondent and administrative) and cultural/language adaptations – are addressed in earlier project stages (see above).

**Table 3.** Measurement properties are to be examined in the validity study.

Reliability	*Test–retest and inter-rater*: In the second assessment (planned to be a week after baseline), half of the sample will be randomly allocated to the same classification user (test–retest) and the other half to a different one (inter-rater). Based on earlier studies,^14^ a week appears sufficiently short for preferences and place of care to remain unchanged but we will explore this issue in the pilot and adapt if need be.
Construct validity	*Convergent validity*: Congruence between the preferred and actual place of care and between the preferred and actual place of death (theoretically, these constructs should be highly related) *Discriminant validity*: Comparison of ‘known groups’ from prior research expected to differ in preferences and/or place of death (e.g. children, adolescents, adults; disease groups; patients, carers)
Responsiveness	Changes over time in preferences and places, comparing patients with heart and cerebrovascular diseases (group expected to change) *versus* cancer (group expected to remain more stable)
Data quality	Data quality assessment (e.g. missing data) and test of distribution assumptions, identifying categories with too high or too low frequencies (to consider for category expansion or reduction)
Interpretability	Production of meaningful ‘benchmarks’ to facilitate interpretation of ICP distributions (of preferred and actual places) in the different disease groups stratified by age and gender Relationship between changes in preferences to changes in actual places of care Preference as a predictor of the last relevant event (place of death). This will assign added meaning to the ICP

The findings from this study will allow us to conclude if the ICP can be robustly applied cross-nationally, achieving our second objective of the project.

### Sample size calculations for quantitative studies

There is no agreement on what is the adequate sample size for psychometric studies but a sample of at least 50 participants is adequate for the assessment of agreement (test-retest reliability).^38^ To be able to detect a 20% change in preferences for dying at home (based on earlier indicative findings),^14^ we would need 52 participants (responsiveness to change). To ensure sufficient numbers in each disease group, allowing for some variation, we will therefore include 392 families (98 per country), including 224 families of adult patients (56 per country) and 168 families of children/adolescents (42 per country). These calculations apply also to the longitudinal study.

### Data integration

The third and last objective of the project will be achieved through the integration of the qualitative and quantitative findings from all the studies. The research team will work together in this integration, bringing in different disciplines, expertise, culture and philosophical beliefs, which will enrich the interpretation of the findings. Aligning with a mixed-methods approach, the use of different methods will allow a complete picture of the different preferences, pathways and transitions that are meaningful for individuals at the end of life. We will achieve integration by appraising study findings side-by-side to find out if they converge, depart or complement each other. We will search for inter-method discrepancies that may result in a better understanding of diversity in end-of-life pathways. We will combine several techniques for integrating mixed-methods data^39^; these include (i) visual models following threads between the different studies to help interpret findings that require further exploration, (ii) studying together all the data collected on each single country case and (iii) developing a convergence coding matrix to identify meta-themes across studies and countries.

### Ethics

The study raises several ethical issues as it involves vulnerable individuals, including patients, minors and adults lacking capacity, across EU and non-EU countries. Approvals to conduct the research have been obtained from the ethics committee of the host organization – Faculty of Medicine of the University of Coimbra (068-CE-2022), the Mulago Hospital Ethics and Research Committee (MHREC 2022-70), the Uganda National Council of Science and Technology in Uganda (SS1537ES), the Leiden University Medical Center (non-WMO division 3, nr 22-3074) and the University of Kansas Medical Center (STUDY00150249). The Brown University IRB issued a non-human research determination covering the non-clinical components in the United States. We will also seek approval from ethics committees of other clinical recruitment sites for the clinical component that takes part in their centre. Providers of individual anonymized death certificate data from Portugal, the Netherlands and Uganda have authorized access to these data for research purposes. For the United States, we are accessing individual anonymized public-use files, made available free of charge by the Centers for Disease Control and Prevention (CDC) through their website. Informed consent will be obtained from study participants or their legal representatives in case of minors and adults lacking the capacity, to comply with local law (procedures described later). Although our research does not involve interventions or changes to usual care, it takes time and effort from patients and their family carers who at some point experience and have to deal with distress and problems related to a naturally advancing illness. Their participation is important not only to ensure methodological thoroughness and generalizability of findings but also to ensure the new ICP is truly built on their experiences and perspectives. The research will be conducted by a team trained in ethics specifically applied to the project and in compliance with the ethical standards and guidelines of H2020, the MORECare guidance on ethical issues in palliative and end-of-life care research,^40^ the Declaration of Helsinki and the Oviedo Convention, the General Data Protection Regulation (GDPR) and any applicable national or state laws applicable in fieldwork sites. Local cultural sensitivities will also be respected. Careful consideration has been given to the risks of harm based on the successful experience of conducting research at the end of life, established guidelines^40–43^ and studies showing benefits from end-of-life care research.^44–46^ Measures to address the risks of harm will include the following: patients and carers will be recruited by care staff to avoid approaching patients and carers who are not suitable for the study or are most vulnerable (too ill, too stressed, too overwhelmed); detailed procedures for obtaining written informed consent guaranteeing confidentiality within legal and ethical limits and the security of personal data, and enabling the participation of illiterate persons (through the involvement of a witness); use of materials and vocabulary that lay friendly and easy to understand; serial interviews *via* telephone calls when possible to minimize disruption of daily routines; researchers trained to be attentive to any expression of discomfort/distress and to inform participants of local sources of help should this be required; distress protocol in place to handle distress events, complaints or other situations of the high level of concern that may require action. In the ethnography study, we will use non-participant observation, which involves overt observation of patients, family carers and care staff in different caring environments without the researcher’s active participation. Care staff will be informed about our presence. In the beginning phase, we will carry out broad-scope observation and allow sufficient time for the care staff, patients and family carers to feel comfortable with our presence. This will be followed by a more focused observation where we will pay attention to narrower activities within care environments. We will sample different time periods to capture variation and avoid prolonged or unnecessary observation time. We will apply a series of measures to increase trust with the people we will be observing by non-intrusive presence, attuning to body language and communication patterns as cues about discomfort, taking account of matters of safety, feelings of being observed which may manifest as discomfort, social and cultural context, values and beliefs. The researchers will take detailed fieldnotes (no visual recorders/cameras will be used). We do not intend to make identified references to a person’s behaviour; we will observe only public expressions and actions that will not identify an individual. We have put in place special procedures for minors and adults lacking the capacity to provide consent complying with local law. In Portugal, we will obtain informed consent from the parents/legal representatives for minors less than 6 years of age, consent from parents/legal representatives and assent from minors with 6–15 years of age (adapted to different ages and maturity, and in writing for those aged 11–15), and consent of parents/legal representatives and consent from minors with ages 16 or 17. In Uganda, complying with national law on age for assent and consent, we will seek assent from children aged 8–17 and consent from their parents. In the Netherlands, we will seek assent from children/adolescents aged 6–15 and consent from their parents/legal representatives, and consent of adolescents from 16 onwards, provided their parents/legal representatives’ assent for him/her to take part. In the United States, we will seek assent from children aged 6–17 and consent from their parents/legal representatives. We will only obtain data on place of care but not place of death for younger children aged 6–15 years because it is a sensitive topic. Data on the preferences of minors who are sufficiently cognitively and emotionally mature to express their preferences are particularly valuable, but this will need to balance between protecting participants and doing what is in their best interests as a group. Different versions of consent/assent forms have been developed and adapted to different ages (for children) and levels of maturity/capacity (e.g. adapting forms using pictures/symbols when asked to indicate their preferred involvement). Depending on the patient’s reading and comprehension ability, the form will be read to or with them. They will have the opportunity to ask questions and the researcher will explain anything that is not clear. Even when the parents/legal representatives provide consent, if the patient can formulate an opinion and evaluate the information and express a wish not to take part, the research team will respect their wish. A similar approach will be taken with adults lacking the capacity to provide consent – we will obtain informed consent from their legal representative, which should reflect the presumed wish of the patient; the patient will be provided information about the study (including its risks and benefits) which is adequate to their level of comprehension; and we will consider the expressed wish of the patient. We will assess capacity following successful practice in a recent study on end-of-life care with patients with dementia (EMBED-CARE),^47^ trained local clinical staff or a member of the research team will conduct a brief structured assessment of capacity to consent based on the following criteria: a person is unable to decide for him/herself if they are unable to (1) understand the information relevant to the decision, (2) retain that information, (3) use or weigh that information as part of the process of making the decision or (4) communicate their decision (whether by talking, using sign language or any other means). Prior to fieldwork, the research team will be trained in legal and ethical aspects specific to end-of-life research and to the project, including the procedures to determine the capacity to provide informed consent with a focus on minors and adults with different levels of cognitive impairment. We will ensure a sensitive approach to recruitment that demonstrates empathy, is responsive to each person’s level of understanding and emphasizes the voluntary nature of participation. All parties will ensure the protection of personal data and will not disclose identifiable information, except when required by law or in case of a high level of concern with a person’s well-being. Personal information will be kept secure in a ‘study codebook’, separate from pseudonymized data. Interview and focus group recordings will be destroyed after quality checks have been performed in transcripts (physical destruction of hard drives). As soon as data collection finishes, we will transfer all other raw data to the University of Coimbra (host institution) for archiving, ensuring all data transfers comply with the laws of the country in which the data are collected and with local authorizations for export, according to a tailored data transfer agreement. Personal data will be retained for 7 years after the study ends to allow time for audit and checks until adequate dissemination. After this period, the principal investigator will destroy all personal data. The resulting anonymized data will be kept to allow further analysis and merged analysis with sibling studies if conducted in other countries. The proposed research involves fieldwork in a low-income country (Uganda). The highest ethical standards will be applied in all countries and Uganda is no exception. The project will be conducted in collaboration with local stakeholders, in line with and responsive to local research needs and priorities. It will also involve an element of capacity building, supporting local researchers to participate in the study and providing mentorship as appropriate. Ethical approval has been granted from the appropriate review bodies and local ownership of the data will be assured along with authorship of any materials resulting from the study when applicable and with the involvement of in-country researchers. The results of the research will be shared in-country and it is anticipated that the ICP will apply to and be utilized in the variety of countries involved, including Uganda and other low-income countries. Dissemination will take place at the national as well as international levels involving key local and international stakeholders. There are no foreseen security risks in taking part in research in Uganda, although as in any country, it will be important to assess the situation as the study progresses. A risk assessment plan, which includes a table summarizing the risk assessment plan for the whole project and country-specific risk assessment plan tables (including mitigation measures), has been developed and will be monitored and updated throughout the project aligning to changes in the research or any change in the circumstances and the law of the countries involved.

## Discussion

The EOLinPLACE project will unpick the dynamics and diversity of preferences and places where people are cared for at the end of life and will lead the way in the reform of classifications of dying places which are currently incomplete and inconsistent. Systems such as the International Classification of Diseases (ICD), the Diagnostic and Statistical Manual of Mental Disorders (DSM) and the International Classification of Functioning, Disability and Health (ICF) are examples that have greatly helped monitor the incidence and prevalence of diseases and health issues, identify risk factors, reduce inequities, inform management and funding and improve care worldwide.

An ICP would have a smaller set of categories than the above-mentioned classifications but this would capture nearly all of the variation observed in dying places and in addition, it increases the chances of the ICP being used in practice. The ICP will enable key developments such as the measurement of progress towards achievement of preferences when care can be planned. It can flag situations of concern (e.g. hospitals with a high percentage of people dying in corridors) and map mortality trends in high-tech environments (e.g. ICU) or for certain groups (e.g. psychiatric facilities, prisons). It can quantify the number of people dying in critical places for ageing societies (e.g. care homes) and paediatrics (e.g. hospital isolation rooms). It can also help better control sudden causes by knowing their location (e.g. patterns of ambulance death in myocardial infarction). The potential value to quantify and qualify death is huge and covers all humans. It can change death records and improve research and practice.

The endeavour has challenges, as the metric is more prone to international variation than those of other health-related classifications therefore requiring a general framework that takes into account each national context and network of influencing factors. We will also face well-known challenges in conducting palliative and end-of-life research, for example in the recruitment and follow-up of our target groups, in particular of children and their families. The involvement of an expert advisor on paediatric palliative care (who is helping train the team and will provide advice throughout the project) will help us handle and seek solutions that we hope to share with others to help advance the field.

Reforming the way dying places are classified and understood can only be done by cross-cutting research fields from social and health sciences, working alongside patient and carer representatives. By bringing together novel qualitative and quantitative insights through mixed methods, we will construct higher-level knowledge to better discern and understand the diversity in individual end-of-life pathways. The funding by the European Research Council granted us the talented human resources and the time to develop a robust ICP that can be applied in goal setting, care planning, monitoring and outcome measurement to help people be cared for and die in their place of choice. It will enable the very exciting possibility of pursuing a breakthrough development in this important universal theme, leading to new scientific discoveries, opening new directions in how to care for the dying, with high scientific impact and far-reaching implications, from public health to demography, education and economics.

## Declarations

**Ethics approval and consent to participate:** Detailed information regarding ethics approvals and consent to participate is provided in the Methods section.

**Consent for publication:** Not applicable.

**Availability of data and materials:** Not applicable.
